# Biocatalyzed Synthesis of Glycostructures with Anti-infective Activity

**DOI:** 10.1021/acs.accounts.2c00136

**Published:** 2022-08-09

**Authors:** Pilar Hoyos, Almudena Perona, Teodora Bavaro, Francesca Berini, Flavia Marinelli, Marco Terreni, María J. Hernáiz

**Affiliations:** † Departamento de Química en Ciencias Farmacéuticas, Facultad de Farmacia, Universidad Complutense de Madrid, Plaza Ramón y Cajal s/n, 28040 Madrid, Spain; ‡ Dipartimento di Scienze del Farmaco, Università di Pavia, Viale Taramelli 12, 27100 Pavia, Italy; § Dipartimento di Biotecnologie e Scienze della Vita, Università degli Studi dell’Insubria, Via Dunant 3, 21100 Varese, Italy

## Abstract

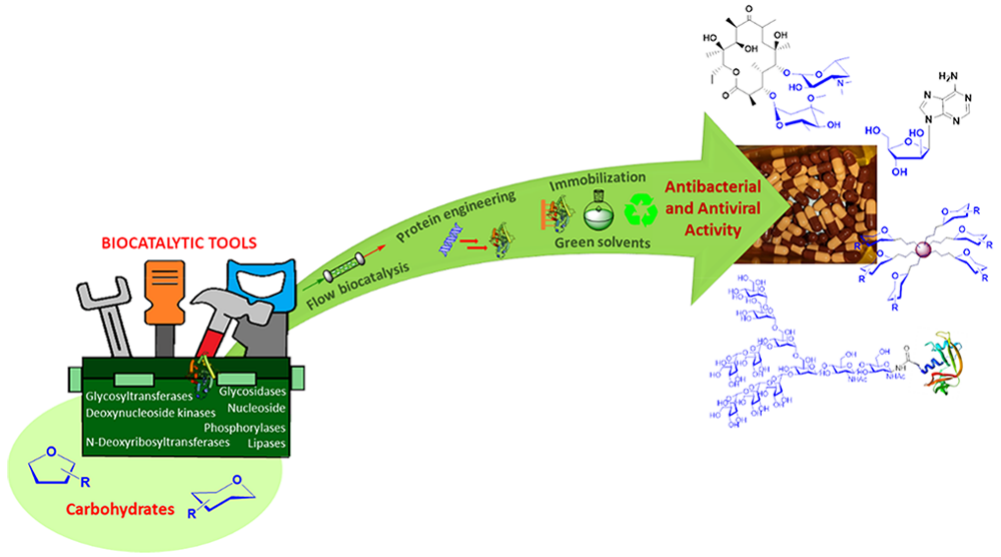

Molecules containing carbohydrate moieties play essential roles in fighting a variety of bacterial and viral infections. Consequently, the design of new carbohydrate-containing drugs or vaccines has attracted great attention in recent years as means to target several infectious diseases.

Conventional methods to produce these compounds face numerous challenges because their current production technology is based on chemical synthesis, which often requires several steps and uses environmentally unfriendly reactants, contaminant solvents, and inefficient protocols. The search for sustainable processes such as the use of biocatalysts and eco-friendly solvents is of vital importance. Therefore, their use in a variety of reactions leading to the production of pharmaceuticals has increased exponentially in the last years, fueled by recent advances in protein engineering, enzyme directed evolution, combinatorial biosynthesis, immobilization techniques, and flow biocatalysis. In glycochemistry and glycobiology, enzymes belonging to the families of glycosidases, glycosyltransferases (Gtfs), lipases, and, in the case of nucleoside and nucleotide analogues, also nucleoside phosphorylases (NPs) are the preferred choices as catalysts.

In this Account, on the basis of our expertise, we will discuss the recent biocatalytic and sustainable approaches that have been employed to synthesize carbohydrate-based drugs, ranging from antiviral nucleosides and nucleotides to antibiotics with antibacterial activity and glycoconjugates such as neoglycoproteins (glycovaccines, GCVs) and glycodendrimers that are considered as very promising tools against viral and bacterial infections.

In the first section, we will report the use of NPs and *N*-deoxyribosyltransferases for the development of transglycosylation processes aimed at the synthesis of nucleoside analogues with antiviral activity. The use of deoxyribonucleoside kinases and hydrolases for the modification of the sugar moiety of nucleosides has been widely investigated.

Next, we will describe the results obtained using enzymes for the chemoenzymatic synthesis of glycoconjugates such as GCVs and glycodendrimers with antibacterial and antiviral activity. In this context, the search for efficient enzymatic syntheses represents an excellent strategy to produce structure-defined antigenic or immunogenic oligosaccharide analogues with high purity. Lipases, glycosidases, and Gtfs have been used for their preparation.

Interestingly, many authors have proposed the use Gtfs originating from the biosynthesis of natural glycosylated antibiotics such as glycopeptides, macrolides, and aminoglycosides. These have been used in the chemoenzymatic semisynthesis of novel antibiotic derivatives by modification of the sugar moiety linked to their complex scaffold. These contributions will be described in the last section of this review because of their relevance in the fight against the spreading phenomenon of antibiotic resistance. In this context, the pioneering *in vivo* synthesis of novel derivatives obtained by genetic manipulation of producer strains (combinatorial biosynthesis) will be shortly described as well.

All of these strategies provide a useful and environmentally friendly synthetic toolbox. Likewise, the field represents an illustrative example of how biocatalysis can contribute to the sustainable development of complex glycan-based therapies and how problems derived from the integration of natural tools in synthetic pathways can be efficiently tackled to afford high yields and selectivity. The use of enzymatic synthesis is becoming a reality in the pharmaceutical industry and in drug discovery to rapidly afford collections of new antibacterial or antiviral molecules with improved specificity and better metabolic stability.

## Key References

- RinaldiF.; Fernández-LucasJ.; de la FuenteD.; ZhengC.; BavaroT.; PetersB.; MassoliniG.; AnnunziataF.; ContiP.; de la MataI.; TerreniM.; CalleriE.Immobilized enzyme reactors based on nucleoside phosphorylases and 2′-deoxyribosyltransferase for the in-flow synthesis of pharmaceutically relevant nucleoside analogues. Bioresour. Technol.2020, 307, 1232583224727610.1016/j.biortech.2020.123258.^1^*This article describes the synthesis of nucleoside analogues by immobilized enzyme reactors based on nucleoside 2′-deoxyribosyltransferases and phosphorylases. The developed in-flow system represents an advance in glycochemistry for the preparation of pharmaceutically relevant glycocompounds reported in this Account*.
- LiZ.; BavaroT.; TengattiniS.; BernardiniR.; MatteiM.; AnnunziataF.; ColeR. B.; ZhengC.; SollogoubM.; TamboriniL.; TerreniM.; ZhangY.Chemoenzymatic synthesis of arabinomannan (AM) glycoconjugates as potential vaccines for tuberculosis. Eur. J. Med. Chem.2020, 204, 1125783271748210.1016/j.ejmech.2020.112578.^2^*This article reports the chemoenzymatic synthesis of branched oligosaccharide mimetics of the arabinomannan antigen of *Mycobacterium tuberculosis*. This work represents progress in the preparation of a new glycoconjugate vaccine against tuberculosis, one of the topics described in this Account.*
- HoyosP.; BavaroT.; PeronaA.; RumberoA.; TengattiniS.; TerreniM.; HernáizM. J.Highly efficient and sustainable synthesis of neoglycoproteins using galactosidases. ACS Sustainable Chem. Eng.2020, 8, 6282–6292.^3^*This article describes an innovative method for the chemoenzymatic synthesis of neoglycoproteins based on the use of glycosidases in green solvents. This work opens a new, simple, and green pathway for the synthesis of relevant glycostructures such as vaccines*.
- YushchukO.; ViorN. M.; Andreo-VidalA.; BeriniF.; RuckertC.; BuscheT.; BindaE.; KalinowskiJ.; TrumanA. W.; MarinelliF.Genomic-led discovery of a novel glycopeptide antibiotic by *Nonomuraea coxensis* DSM 45129. ACS Chem. Biol.2021, 16, 915–9283391370110.1021/acschembio.1c00170PMC8291499.^4^*This article deals with the genome-guided discovery of a novel glycopeptide antibiotic from a rare actinomycete, with a particular focus on the description of the enzymes involved in sugar modification of the aglycone core*.

## Introduction

Carbohydrate-containing molecules play a relevant role in contrasting infectious diseases,^5,6^ which are among the most recurrent causes of morbidity and mortality because of the current paucity of efficient drugs or vaccines. Numerous antibacterial and antiviral drugs are derived from or contain a carbohydrate moiety, which contributes substantially to their biological activity.^7^ This is the case of glycopeptide antibiotics,^8^ macrolides,^9^ aminoglycosides,^10^ nucleosides,^1^ neuraminidase inhibitors,^11^ and GCVs,^6,12^ which have different (bio)synthetic origins and modes of action. Carbohydrates are typically complex and heterogeneous in nature, and their synthesis can be challenging, being traditionally based on chemical approaches using toxic reagents and inefficient protocols. Developing biocatalysts and eco-friendly solvents is of vital importance for their sustainable production in the pharmaceutical industry.^13,14^ In this context, the biocatalysts used most are enzymes belonging to the families of glycosidases, glycosyltransferases (Gtfs), lipases, and NPs.^15^ Their use in a variety of reactions for the synthesis and derivatization of carbohydrates has increased exponentially in the last years, favored by the recent advances in immobilization techniques,^16−19^ protein engineering,^20^ enzyme directed evolution,^21^ combinatorial biosynthesis, the use of green solvents,^13,22−31^ and flow biocatalysis.^32^

The intrinsic pharmacokinetic properties of carbohydrates due to their hydrophilic nature and their short serum half-lives have delayed their exploitation in medicinal chemistry as a source of new drugs. Most of these drawbacks have recently started to be addressed with the rational design of glycomimetics with improved pharmacokinetic properties. As carbohydrates are easily degraded, hydrolysis can be minimized by replacing the *O*-glycoside atom with a more stable surrogate atom (*e.g.*, C or S), using biomimetic groups to replace functional groups that are associated with rapid hydrolysis, or adding electronegative substituents to the glycan ring to destabilize the oxocarbenium transition state required for degradation.^33^ Because of their polarity, carbohydrates are poorly absorbed into circulation. To solve this problem, prodrugs in which polar functionalities are temporarily masked by hydrophobic groups (which will be then cleaved by endogenous enzymes after uptake into circulation) have been proposed. Actually, biocatalysis is increasingly employed in the synthesis of new glycomimetic and carbohydrate drugs^1−4,34^ to be produced in large quantities for in-depth structure–activity relationship studies, which are crucial for drug discovery processes. Additionally, biocatalysis fits perfectly with the principles driving the green and sustainable chemistry revolution. The excellent chemo-, regio-, and stereoselectivity displayed by enzymes and the mild reaction conditions required are very interesting features for pharmaceutical production. Enzyme-based procedures might be highly efficient and economically sustainable, and they generate less waste than conventional organic synthesis.^35^

**Scheme 1:**
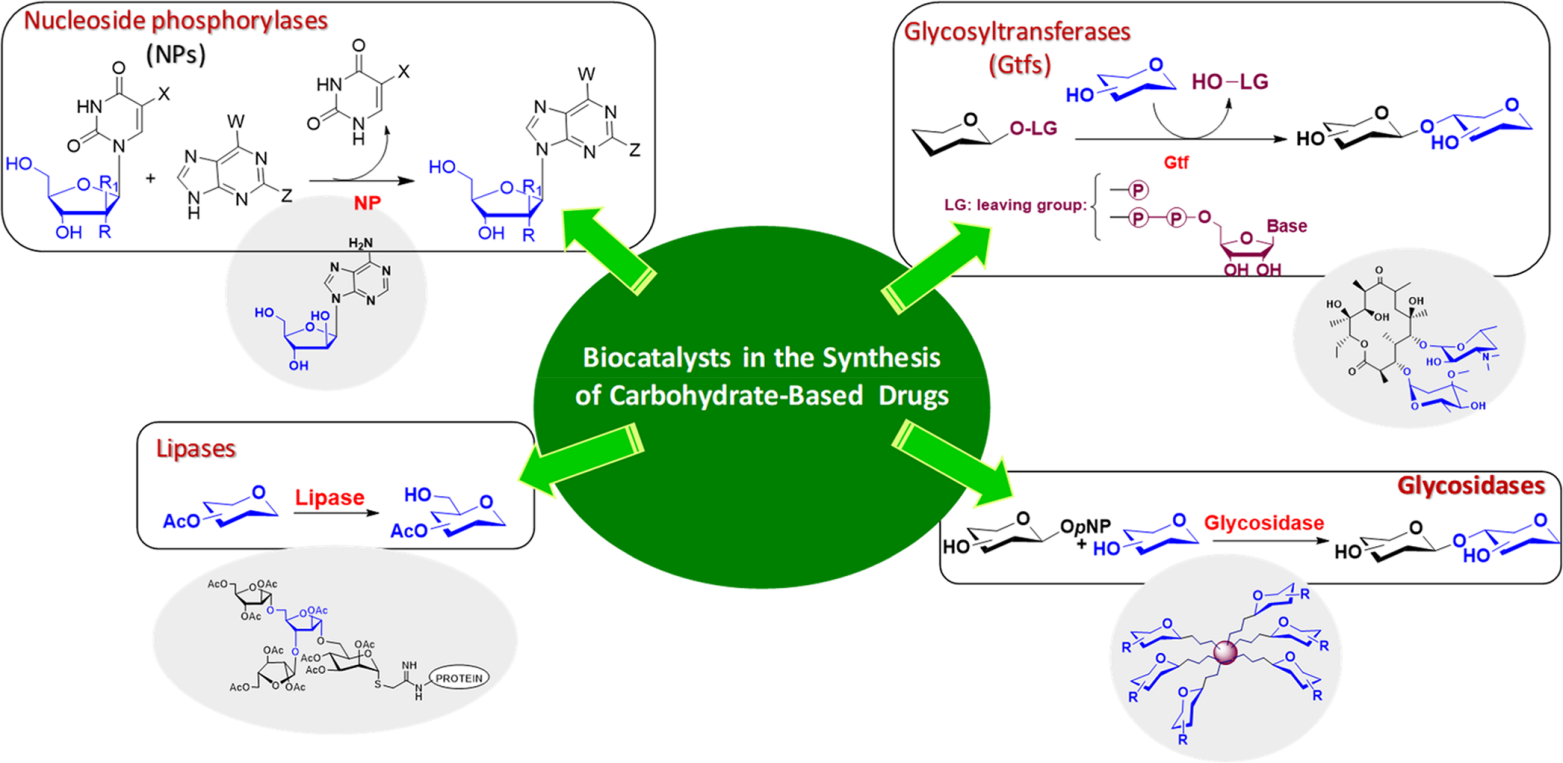
Biocatalytic Methods for the Synthesis of Glycostructures with Anti-infective Activity

Our research groups have been involved for years in various projects for the development of biocatalysts and bioprocesses aiming at the synthesis of compounds of pharmaceutical interest ranging from antiviral nucleosides and nucleotides to glycoconjugated products such as GCVs, glycodendrimers, and glycopeptide antibiotics (Scheme 1). The aim of this Account is to conceptualize the use of biocatalysts in the sustainable synthesis of carbohydrate-based drugs by critically discussing the most interesting strategies based on our research experiences. A whole-picture perspective will be provided through proof-of-concept approaches that combine biocatalysis and carbohydrates.

## Biocatalyzed Synthesis of Nucleosides and Nucleotides with Antiviral Activity

### Biocatalyzed Synthesis of Nucleoside Analogues using NPs and *N*-Deoxyribosyltransferases

Nucleoside analogues (NAs) are used as therapeutic tools against several infections, such as hepatitis B virus (HBV), human immunodeficiency virus (HIV), and SARS and MERS coronaviruses, among others. Thus, antiviral agents with improved bioavailability and reduced side effects have been extensively studied.^36,37^

**Scheme 2:**
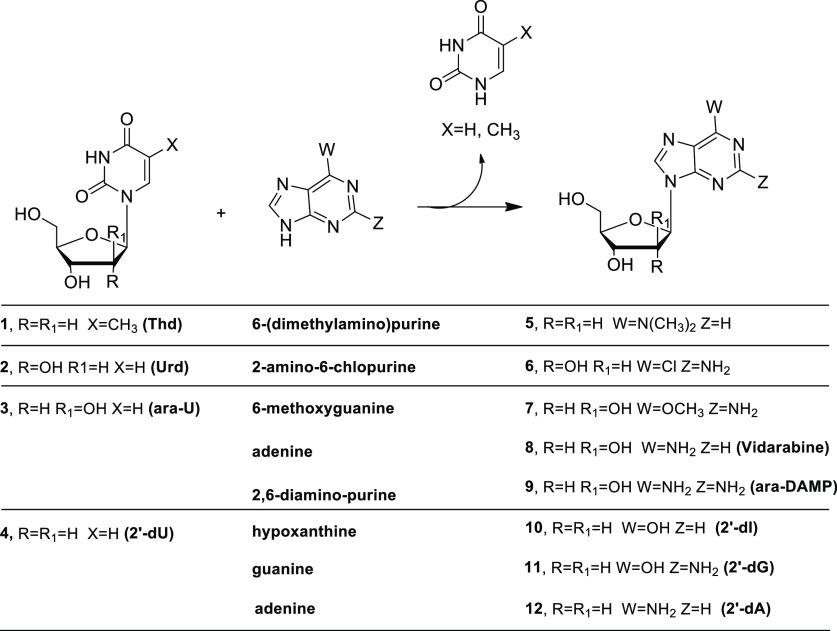
Synthesis of 2′-Deoxyribonucleoside Analogues Catalyzed by UP or TP and PNP

NPs have been investigated for the development of efficient syntheses of non-natural nucleosides (Scheme 2) in aqueous media without the use of protecting groups. We have been involved for several years in the investigation of biocatalysts based on immobilization of NPs such as uridine phosphorylase (UP), thymidine phosphorylase (TP), and purine nucleoside phosphorylase (PNP).

**Scheme 3:**
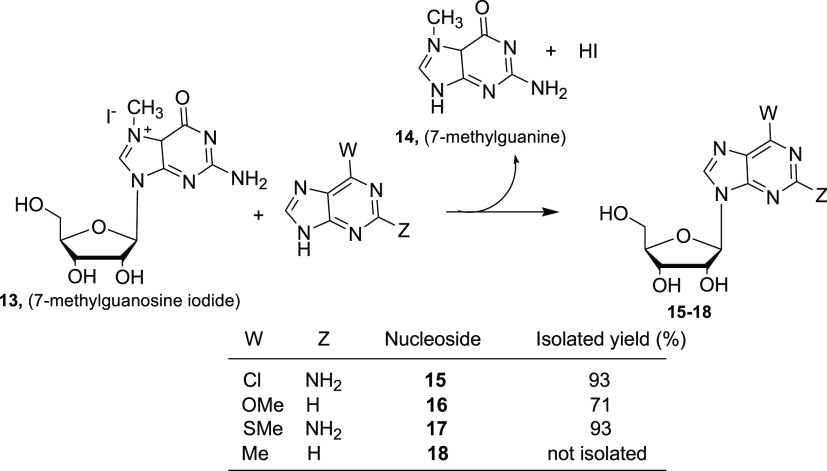
Synthesis of 6-Substituted Ribonucleosides Catalyzed by *Ah*PNP

For example, UP from *Bacillus subtilis* was combined with PNP from *Aeromonas hydrophila* (*Ah*PNP) to catalyze sugar transfer from a pyrimidine nucleoside donor to purine base acceptors. Accordingly, these recombinant enzymes have been successfully applied to the synthesis of 2′-deoxyinosine (**10**) in 85% yield and 2′-deoxyguanosine (**11**) in 95% yield (Scheme 2) by a “one-pot, two-enzyme” transglycosylation.^38^ In order to have biocatalysts that are stable under the reaction conditions, we designed a new immobilization strategy based on ionic adsorption onto epoxy resins derivatized with poly(ethylenimine) (PEI) followed by chemical cross-linking with tailor-made aldehyde-dextran to stabilize and prevent UP and PNP desorption.^39^ The substrate specificity of *Ah*PNP was investigated in detail: 1-, 2-, 6-, and 7-modified nucleosides were accepted as substrates, whereas 8-substituted nucleosides were not. Moreover, *Ah*PNP performed the synthesis of **15**–**18** (Scheme 3) by a transglycosylation reaction in very high yields.^40^

**Scheme 4:**
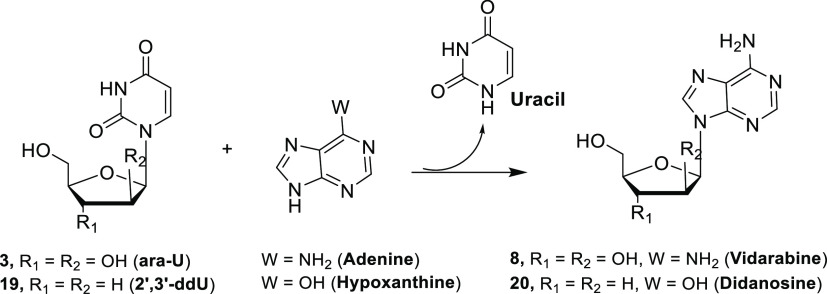
Synthesis of **8** and **20** Catalyzed by NPs

Recently, a new green synthesis of the anti-herpes simplex virus agent vidarabine (**8**) with high purity catalyzed by immobilized NPs (UP from *Clostridium perfringens*, (*Cp*UP) and *Ah*PNP) was developed (Scheme 4).^41^ In fact, *Ah*PNP or PNP from *Citrobacter koseri* in combination with *Cp*UP was used for the synthesis of **8** and the anti-HIV agent didanosine (**20**), achieving 74% and 44% conversion, respectively (Scheme 4).^42^ Biotransformations of 6-substituted ribonucleosides in flow mode using *Ah*PNP immobilized enzyme reactors (IMERs)^32^ turned out to be efficient and fast synthetic processes in which sample handling was minimized.^43^

**Scheme 5:**
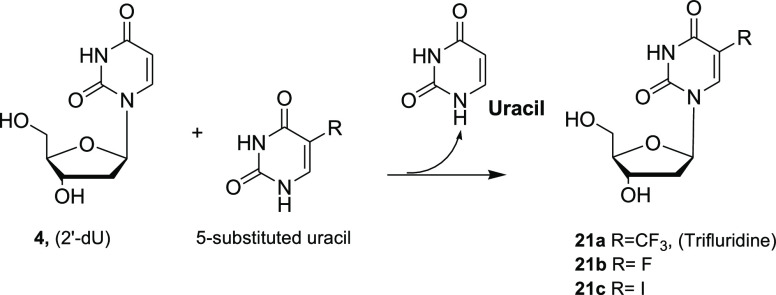
Synthesis of 2′-Deoxyuridine Derivatives Catalyzed by Immobilized NDT

*N*-Deoxyribosyltransferases (NDTs) can provide an alternative to the enzymatic preparation of nucleosides, as these enzymes are suitable to catalyze transglycosylation from a nucleoside sugar donor to both purine and pyrimidine acceptors using the same enzyme, but the strict selectivity for the sugar moiety is a limiting factor for their use. NDT from *Bacillus psychrosaccharolyticus* was immobilized on PEI-coated agarose followed by cross-linking with oxidized dextran and then employed in the synthesis of 2′-deoxyadenosine (**12**) from 2′-deoxyuridine (**4**).^44,45^ The same biocatalyst afforded good yields of the antiviral agent trifluridine (**21a**) on a preparative scale starting from **4** as the sugar donor and 5-trifluorothymine as the base acceptor (Scheme 5).^45^

We recently investigated IMERs based on the immobilization of 2′-NDT from *Lactobacillus reuteri* in flow processes for the synthesis of different NAs by pyrimidine–purine and pyrimidine–pyrimidine transglycosylation, including the synthesis of 5-substituted uridine derivatives such as 5-fluoro- and 5-iodo-2′-deoxyribosyluridine derivatives **21b** and **21c** (Scheme 5).^1^ This enzyme is an efficient tool for the synthesis of 2′-deoxynucleosides that is complementary to the use of NPs, which showed better performance in the synthesis of arabino-nucleosides.^1^

### Biocatalyzed Synthesis of Nucleotide Analogues using Deoxynucleoside Kinases

Deoxynucleoside kinases (DNKs) are the most investigated enzymes for the synthesis of phosphorylated nucleosides. Selective phosphorylation of nucleosides is a challenging process for two reasons: first, the regioselectivity of classical chemical procedures, and second, because the nucleoside analogues as therapeutic agents have to be converted into the corresponding phosphates to exert their pharmacological activity. Thus, we have investigated DNKs for the synthesis of phosphorylated nucleosides used as prodrugs. The multisubstrate DNK from the fruit fly *Drosophila melanogaster* (*Dm*DNK) was first studied in the selective 5′-phosphorylation of natural and non-natural nucleosides. For example, we successfully used immobilized *Dm*DNK for the preparative synthesis of the antiherpes prodrug vidarabine monophosphate (vidarabine-MP, **22**).^46^

Recently, DNK from *Dyctiostelium discoideum* (*Dd*DAK) proved to be a good biocatalyst for the synthesis of adenine 5′-arabinonucleotides with high conversion rates. Differences in substrate specificity of *Dm*DNK and *Dd*DAK make these enzymes complementary to each other. Other authors have investigated the human deoxycytidine kinase for the synthesis of phosphorylated nucleosides, as this enzyme is active on a wide range of natural and modified nucleosides, except for thymidine and uridine derivatives.^47^

**Scheme 6:**
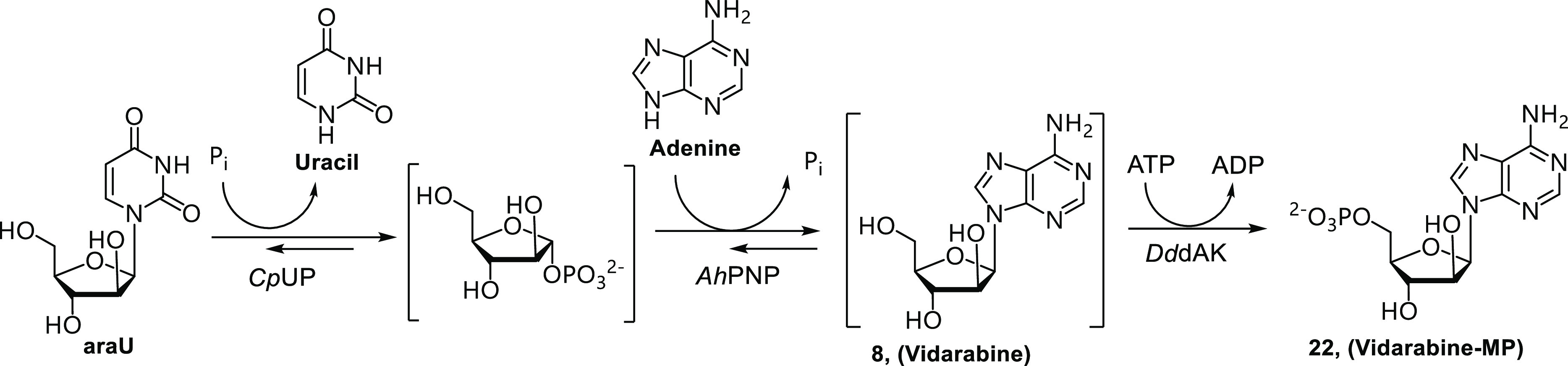
Three-Enzyme Cascade Reaction for the Synthesis of **22**

Along these lines, we investigated the combination of NPs and DNKs to develop a multienzymatic one-pot cascade reaction for the synthesis of **22**.^48^ The selection of the enzymes with appropriate selectivity and the design of tailor-made immobilization protocols were crucial for the process optimization. Specifically, *Cp*UP catalyzed the phosphorolysis of araU, thus generating uracil and α-d-arabinose-1-phosphate, and *Ah*PNP catalyzed the coupling between this latter compound and adenine to form **8**. This nucleoside was the substrate of *Dd*DAK, which selectively produced **22** (Scheme 6), avoiding phosphorylation of the sugar donor araU.

### Biocatalyzed Synthesis of Nucleoside Analogues using Hydrolases

An alternative approach for the synthesis of modified nucleosides is the use of hydrolases for the regioselective hydrolysis of acetylated nucleosides, which provides access to building blocks with a free hydroxyl group at the desired position of the sugar moiety. Interestingly, in this way, modified nucleosides can be prepared and used as donors of non-natural sugars in transglycosylation reactions catalyzed by NPs.^45,49^ In this context, we recently proposed the use of the immobilized protease from *B. subtilis* in the regioselective hydrolysis of several acetylated nucleosides, including antiviral compounds.^50^ This protease can be immobilized by covalent attachment to preactivated supports to yield biocatalysts with good stability.

**Scheme 7:**
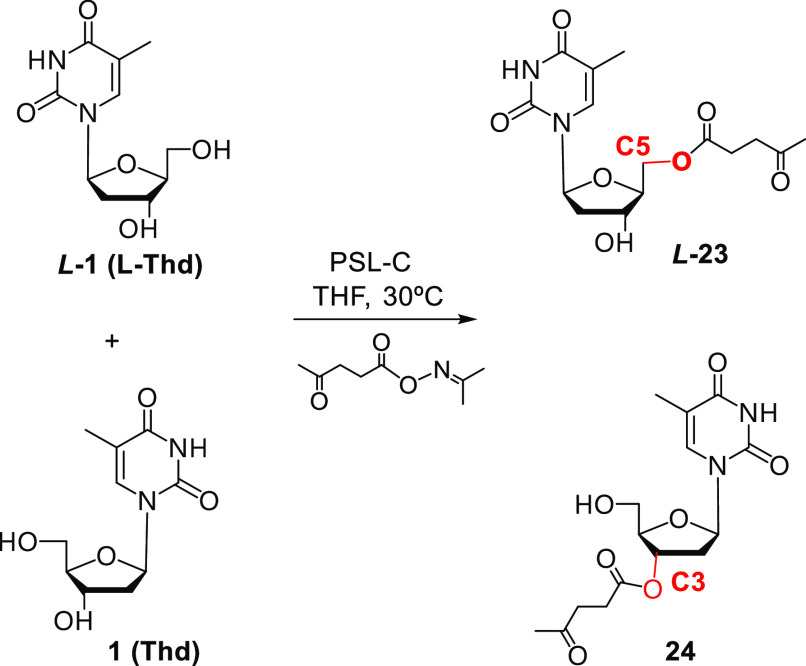
Regioselective Acylation of d/l-Thymidine Catalyzed by PSL-C

Lipases can be also used in acylation reactions performed in organic solvents, and an interesting application was proposed for the preparation of acylated l-nucleosides,^51^ which displayed good therapeutic potential. The synthesis of β-l-5′-*O*-levulinyl-2′-deoxynucleosides was performed through the regioselective esterification of the corresponding l-2′-deoxynucleosides mediated by lipase B from *Candida antarctica* (CALB) or *Pseudomonas cepacia* (PSL-C). This enzyme was also employed in the kinetic resolution of d/l-deoxynucleoside mixtures, as this lipase displays different regioselectivities toward the two isomers (Scheme 7), producing β-l-5′-*O*-levulinylthymidine (l-**23**), an intermediate for the preparation of l-oligonucleotides investigated as therapy against HIV-1 and HBV.

Lipase-mediated acylation of nucleosides has also been performed to increase their lipophilicity. For example, Wang *et al.*(52) described the regioselective acylation of **21a** catalyzed by immobilized PSL-C employing vinyl esters as acyl donors to obtain 3-*O*-acyl derivatives with improved pharmacokinetic properties.

## Chemoenzymatic Synthesis of Glycoconjugates with Antibacterial and Antiviral Activity

**Scheme 8:**
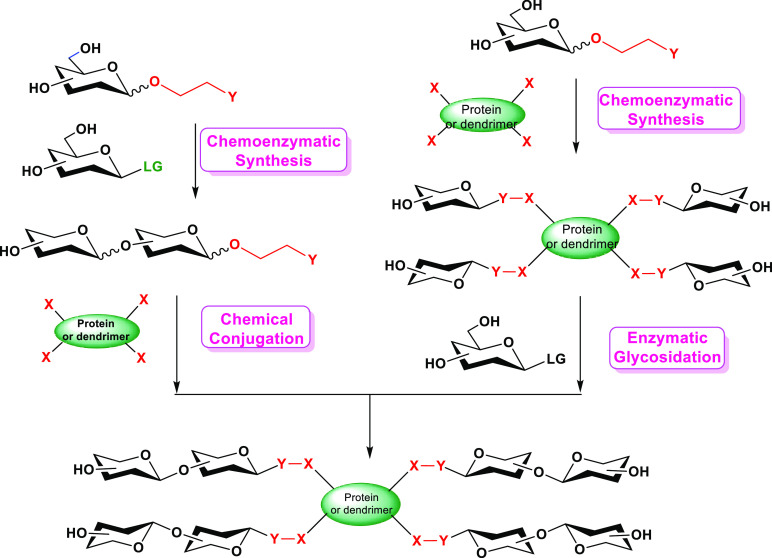
Chemoenzymatic Synthesis of Glycoconjugates (X and Y are Functional Groups)

Carbohydrates conjugated with different macromolecules (glycoconjugates) have been described as important tools to contrast viral or bacterial infection diseases,^6^ with the most important types being GCVs and glycodendrimers.^53,54^ For the development of these glycoconjugates, the combination of chemical and biocatalyzed reactions constitutes an alternative path to produce pure and structure-defined immunogenic oligosaccharides. In this case, the synthesis should be carefully designed in order to obtain the desired oligosaccharides with the right linker and a suitable scaffold (protein or dendrimer) for further conjugation. This chemoenzymatic strategy can be used following two approaches, one that comprises the chemical coupling of an enzymatically glycosylated carbohydrate to a multivalent scaffold and another involving enzymatic glycosylation of the scaffold (Scheme 8).^34,53^

### Glycoconjugates Obtained by Chemical Conjugation of Sugars with Proteins

#### Use of Lipases and Esterases

We have successfully used hydrolases to regioselectively deprotect peracetylated mono- and disaccharides, allowing us to obtain a library of sugar building blocks that are useful as acceptors in chemical glycosylation.^55,56^ Accordingly, taking advantage of the regioselectivity displayed by lipases, we have prepared a series of oligosaccharides with reactive linkers suitable for use as starting materials to obtain semisynthetic glycoproteins (neoglycoproteins).^57^ This chemoenzymatic approach is very versatile and can be used to prepare oligosaccharides with different reactive groups at the anomeric position. Especially, we studied the chemoenzymatic preparation of acetylated oligosaccharides with an anomeric cyanomethylthio group because this linker can be activated to obtain the reactive iminomethoxyethyl (IME) glycan, which selectively reacts with lysine in peptides, avoiding undesirable glycosylation of other amino acid residues and at the same time enabling the creation of a glycomimetic.

**Scheme 9:**
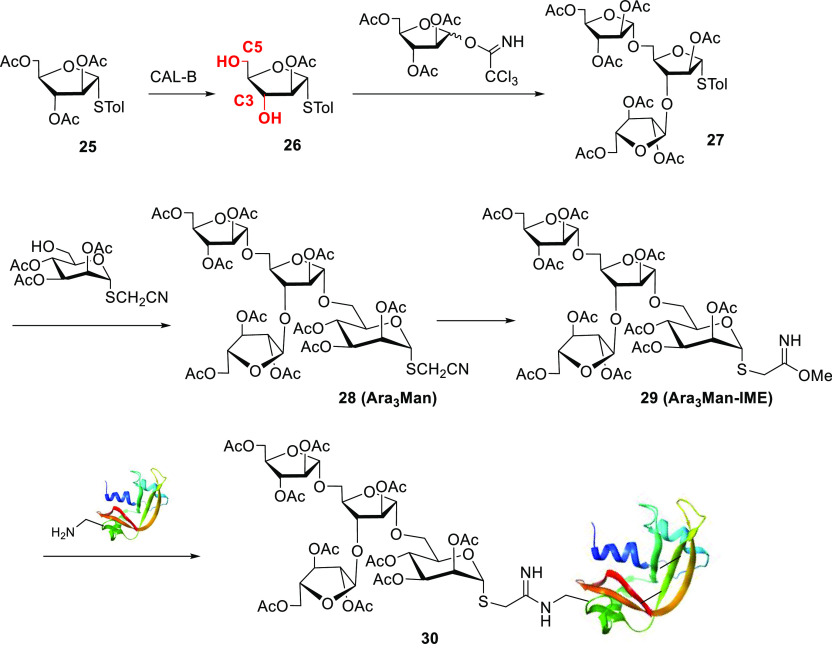
Chemoenzymatic Synthesis of AMM Branched Oligosaccharides and Neoglycoproteins

For instance, immobilized CALB was recently used in a chemoenzymatic synthesis of branched arabinomannan mimetic (AMM) oligosaccharides using a column bioreactor.^2^ Starting from acetylated phenyl thioglycoside **25** (Scheme 9), CALB led to quantitative yields of products hydrolyzed at C5 or both C3 and C5. The latter intermediate, arabinose building block **26**, was used in the chemoenzymatic synthesis of different branched AMMs,^2^ and subsequently, the functionalized IME-glycan **29** was conjugated with human serum albumin (Scheme 9). *Ex vivo* studies of neoglycoprotein **30** revealed a good affinity of the Ara_3_Man group for the antibodies of tuberculosis-infected patients.

**Scheme 10:**
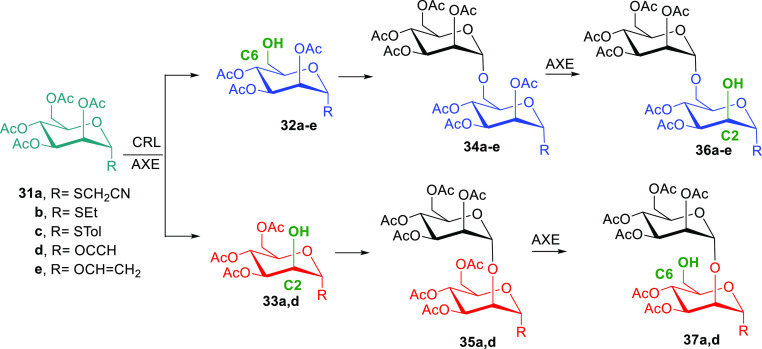
Chemoenzymatic Synthesis of Acetylated Mannose-Based Disaccharides

Similarly, the preparation of mannose glycans was undertaken since these sugars can be used to improve the uptake of antigens by targeting mannose receptors of antigen-presenting cells.^58^ Thus, we performed the synthesis of mannosylated neoglycoproteins *via* the cyanomethylthio group, and the products obtained were tested as potential glycosylated subunits in vaccines against tuberculosis.^59^ Mannose α(1→6) or α(1→2) disaccharides with different anomeric groups were prepared through regioselective hydrolysis of acetylated monosaccharides **31a**–**e** (Scheme 10) catalyzed by immobilized *Candida rugosa* lipase or by acetyl-xylan esterase (AXE) from *Bacillus pumilus*. Next, chemical glycosylation generated disaccharides **34** and **35**, which could be further hydrolyzed by AXE at C2 or C6 of the anomeric mannose.^60^ The adjuvant activity of different mannose oligosaccharides in the preparation of neoglycoproteins is currently under study.

Lipase from *Pseudomonas stutzeri* is a versatile biocatalyst, and we have demonstrated that it can catalyze the regioselective hydrolysis or alcoholysis of a variety of peracetylated monosaccharides in the presence of biomass-derived solvents (biosolvents), particularly glycerol or dimethylamide derivatives. With these biosolvents, the selectivity of the lipase was driven exclusively toward hydrolysis of the acetyl group on the anomeric position of the β anomers, providing a sustainable enzymatic approach to obtain a library of sugar building blocks with a specific configuration of the anomeric position.^56^

#### Use of Glycosidases and Glycosyltransferases

Glycosidases and Gtfs can be used to regio- and stereoselectively synthesize oligosaccharides by glycosylation reactions, fully avoiding the use of protecting groups.

Glycosidases synthesize oligosaccharides in a kinetically controlled manner by the reaction of a glycosyl donor that transfers its glycosyl residue to a sugar acceptor present in the reaction medium. These reactions have been conducted in green solvents, especially ionic liquids or biosolvents because of their multiple advantages, not only from an environmental point of view but also because of their effect on the enzyme performance, varying the enzyme activity and selectivity.^13,23−26,28−31^

In this framework, different β-galactosidases have shown excellent biocatalystic aptitudes for galactosyl transfer from suitable donors onto *N*-acetylglucosamine (GluNAc) as the acceptor when used in green solvents, such as those made from renewable sources that are considered tunable and smart solvents because they can change their properties under different reaction conditions.^22^ In this context, efficient syntheses with very high yields and regioselectivity, avoiding nondesired hydrolytic reactions, have been previously reported for the following glycosylated sugars: Galβ(1→6)GluNAc employing β-galactosidase from *Escherichia coli*(61) or Biolacta (commercial β-galactosidase from *Bacillus circulans*)^24,25^ in combination with glycerol-based biosolvents; Galβ(1→4)GluNAc using β-galactosidase from *Thermus thermophilus* in combination with ionic liquids;^26,27,30^ and Galβ(1→3)GluNAc using β-Gal-3 from *B. circulans* ATCC 31382 in combination with glycerol-based biosolvents^28^ or ionic liquids such as [Bmin][PF_6_].^29^ Furthermore, immobilization of Biolacta on tailor-made porous polymers^17^ and immobilization of β-Gal-3 on a glycosyl–agarose support^18^ provided an effective and sustainable approach to carry out the enzymatic synthesis of these disaccharides, allowing biocatalyst and green solvent recycling.

**Scheme 11:**
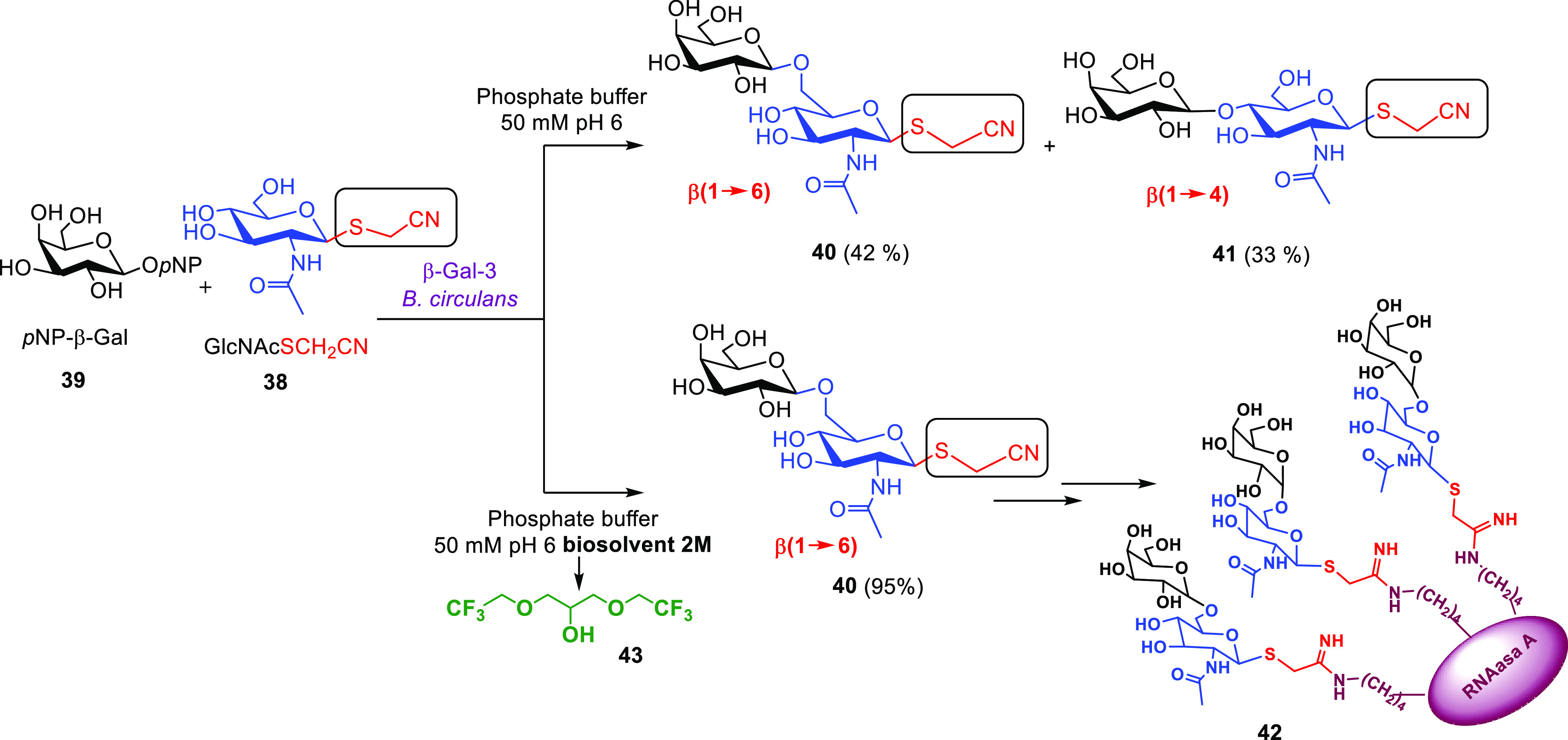
Regioselective Synthesis of Activated Disaccharide **40** Mediated by β-Gal-3 in the Presence of Glycerol-Derived **43**

On the basis of these results, we investigated the use of galactosidases in green solvents for the synthesis of disaccharides containing a cyanomethylthio group at the anomeric position, which were further employed in the preparation of neoglycoproteins.^3^ β-Gal-3 and β-galactosidase from *E. coli* recognized GluNAc functionalized with the cyanomethylthio group at C1 (**38**) as the acceptor (Scheme 11) and activated-galactose derivative **39** as the donor. In the first case, the presence of this activated group induced a change in the substrate recognition pattern of β-Gal-3, affording a mixture β(1→6) and β(1→4) glycosidic linkages instead of the expected β(1→3) linkage. Particularly in this work, the presence of the glycerol-derived solvent **43** (Scheme 11) completely shifted the equilibrium toward the synthesis of the β(1→6) linkage with full regioselectivity. Then the activated disaccharide **40** was successfully employed in the preparation of well-defined neoglycoprotein **42**. As an additional advantage of this sustainable process, the biphasic medium created under these conditions allowed the recycling and reuse of solvent **43**.

**Scheme 12:**
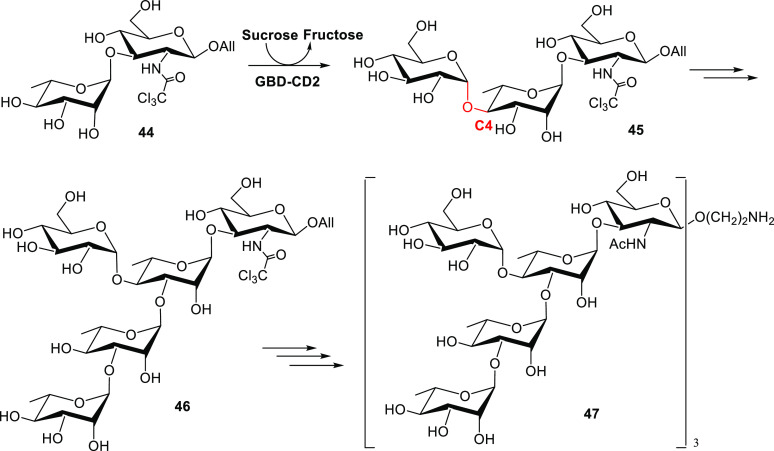
Chemoenzymatic Synthesis of the *Shigella flexneri* Pentasaccharide Repeating Unit

An interesting chemoenzymatic approach involving Gtfs was developed for the preparation of different antigenic oligosaccharides and neoglycoproteins. The α-glucose transferase from *Leuconostoc mesenteroides* was used to obtain a trisaccharide intermediate of the *Shigella flexneri* antigen.^62^ The synthetic disaccharide rhamnose-α-1,3-glucosamine **44** was used as non-natural sugar acceptor of a sucrose-dependent α-transglucosylase for selective α-glycosylation of the rhamnose at C4 (Scheme 12). The product was transformed for the further preparation of **47** (the trimeric form of the *S. flexneri* antigen) bearing the anomeric alkylamino group that was employed for the final protein glycosylation.

**Figure 1:**
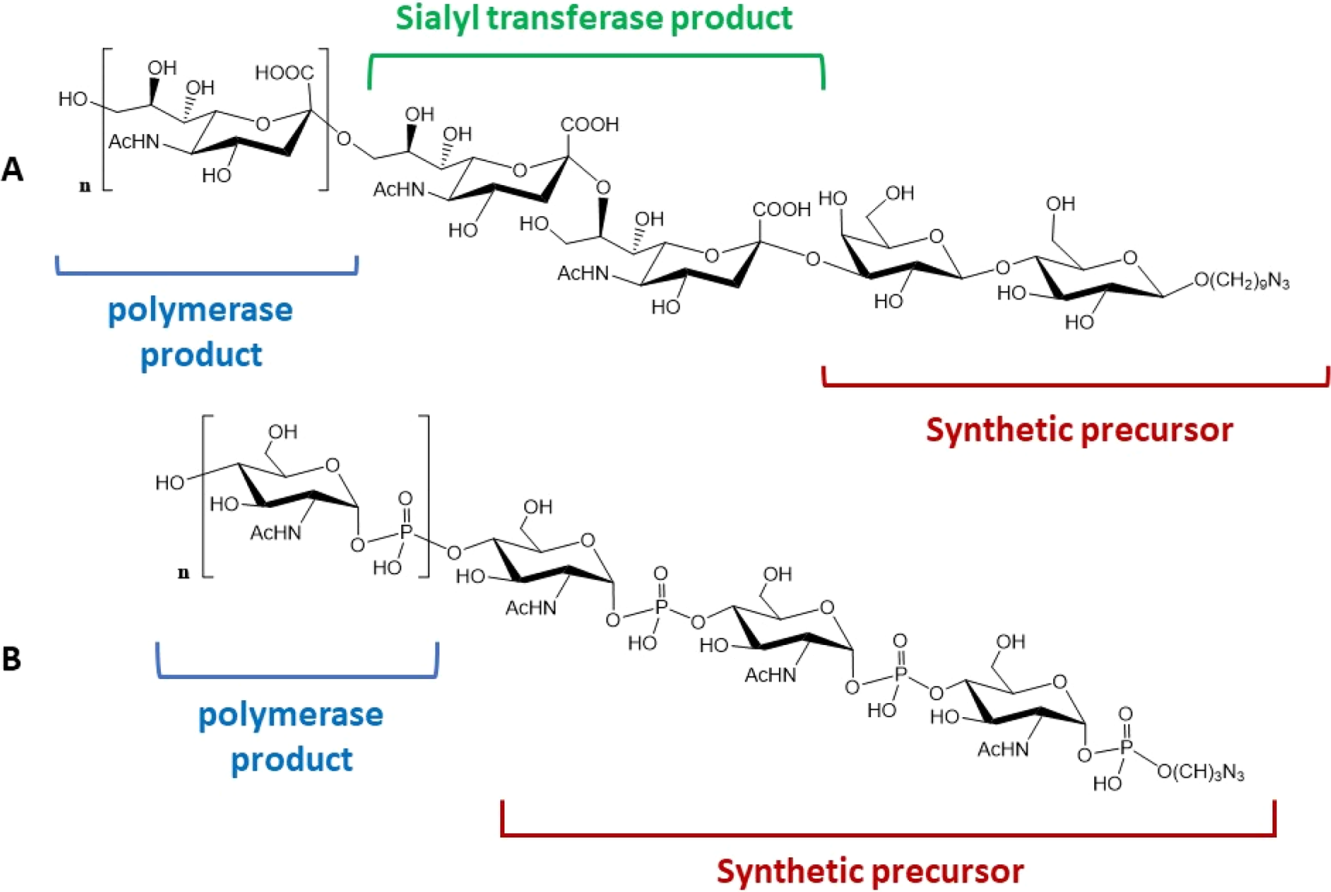
Synthetic antigens of *N. meningitidis* serotypes (A) A and (B) X.

The development of sialyltransferases (STs) obtained from various microbial sources is a front-line technology for the chemoenzymatic synthesis of antigenic oligosaccharides. STs were the starting materials for the synthesis of bacterial antigens, as in the case of *Neisseria meningitidis*. The sugar antigens of *N. meningitidis* are peculiar because of the presence of sialic acid polymers with different glycosidic bonds, except for serotypes A and X, which are not sialylated. The synthesis of antigen-C was achieved by combining two recombinant enzymes, the ST from *Campylobacter jejuni* and the poly-ST of *N. meningitidis* serogroup C. Starting from a synthetic lactose core with an alkylazido linker at the anomeric position, the first enzyme introduced at C3 of the galactose two sialic acids with α(2→8) link. Then the poly-ST performed a chain polymerization that introduced several sialic acid units with an α(2→9) motif (Figure 1A). The oligosaccharide product was conjugated with the immunogenic domain of the *Clostridium tetanus* toxin, and the obtained neoglycoprotein showed immunogenic activity when tested *in vivo*.^63^

Recently, the use of the capsule polymerases (hexose-1-phosphate transferases) from *N. meningitidis* was employed to synthesize non-sialylated *N. meningitidis* serotype A and X antigens, which are composed of *N*-acetylmannosamine or GluNAc, respectively, linked through a phosphodiester bridge.^64^ The synthesis of these polysaccharides was performed using the recombinant sugar polymerases from *N. meningitidis* A and X immobilized in column bioreactors. A chemoenzymatic approach was developed to obtain length-controlled polymers of serogroup X (Figure 1B), performing the on-column enzymatic extension of a chemical precursor composed of three units of GluNAc-1-phosphate and an alkylamino group in the anomeric phosphate.^65^ The product was conjugated with the immunogenic tetanus toxoid variant CRM197, and immunogenic evaluation demonstrated that the fully synthetic GCV had the same activity as the natural oligosaccharide isolated from the pathogen.

### Enzymatic Conjugation of Synthetic Glycans with Scaffolds

**Scheme 13:**
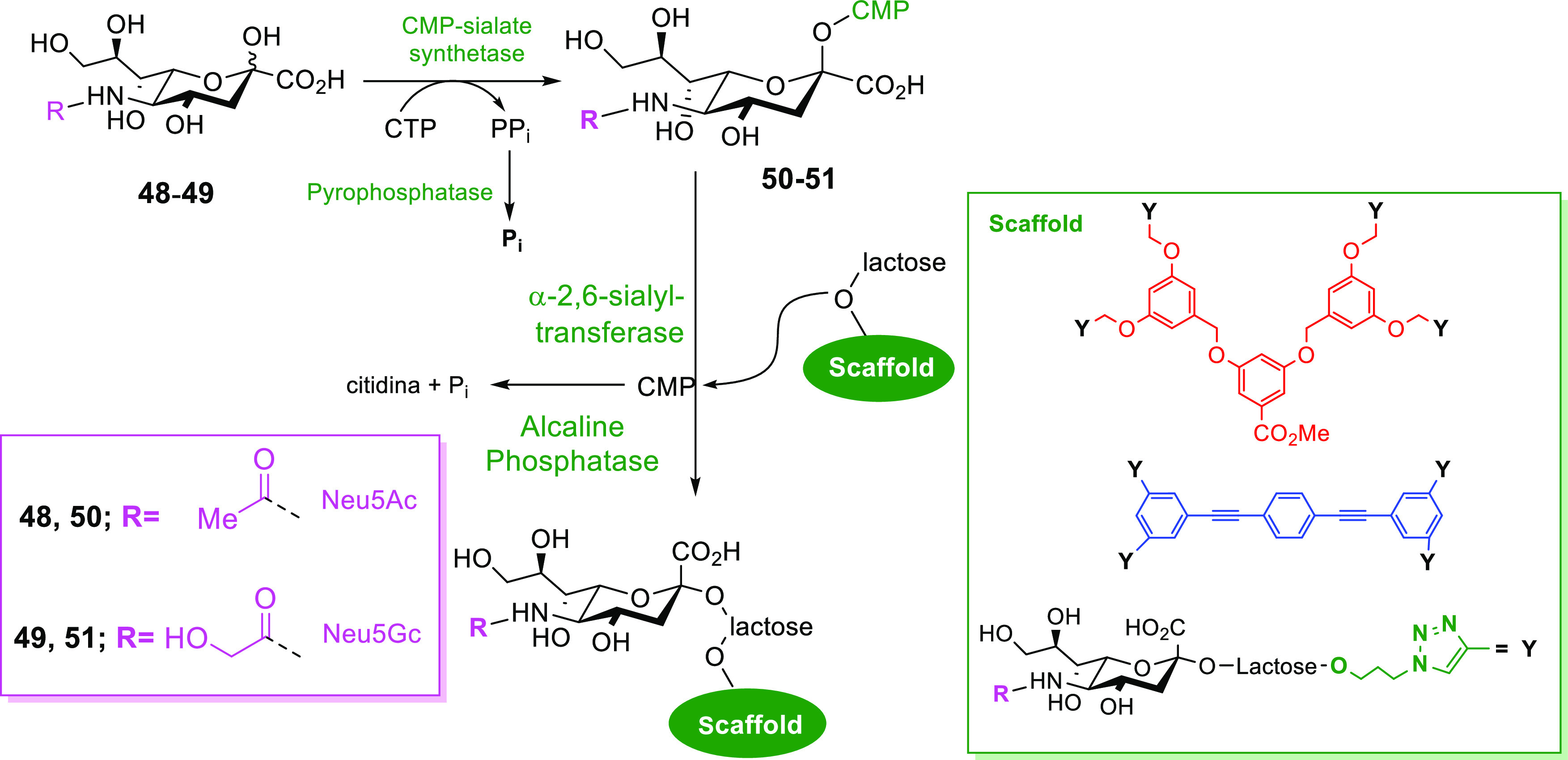
Glycosydation of Glycoclusters Using 2,6-SiaT and Two Different CMP-Activated Sialic Acids as Donors

As mentioned above, an enzymatic strategy can be used that involves direct glycosylation of a simple glycan already attached to a multivalent scaffold (protein or dendrimer). This procedure attracted our attention, and in particular, we illustrated its feasibility by the efficient enzymatic sialylation of a small library of multivalent lactosylated scaffolds by the use of a bacterial α-2,6-sialyltransferase (2,6-SiaT) and two naturally occurring neuraminic acid derivatives. This chemoenzymatic synthesis was executed in three stages: (i) the chemical synthesis of the multivalent core scaffolds, (ii) decoration with β-lactoside units by the use of click chemistry, and (iii) glycan extension by enzymatic sialylation (Scheme 13). A large number of viruses have been shown to interact with sialic acids for cellular attachment and entry, and therefore, entry inhibitors such as glycodendrimers presenting neuraminic acid derivatives could prevent virus attachment to sialic acids and would have the potential to serve as broad-spectrum antiviral drugs.^66^

**Scheme 14:**
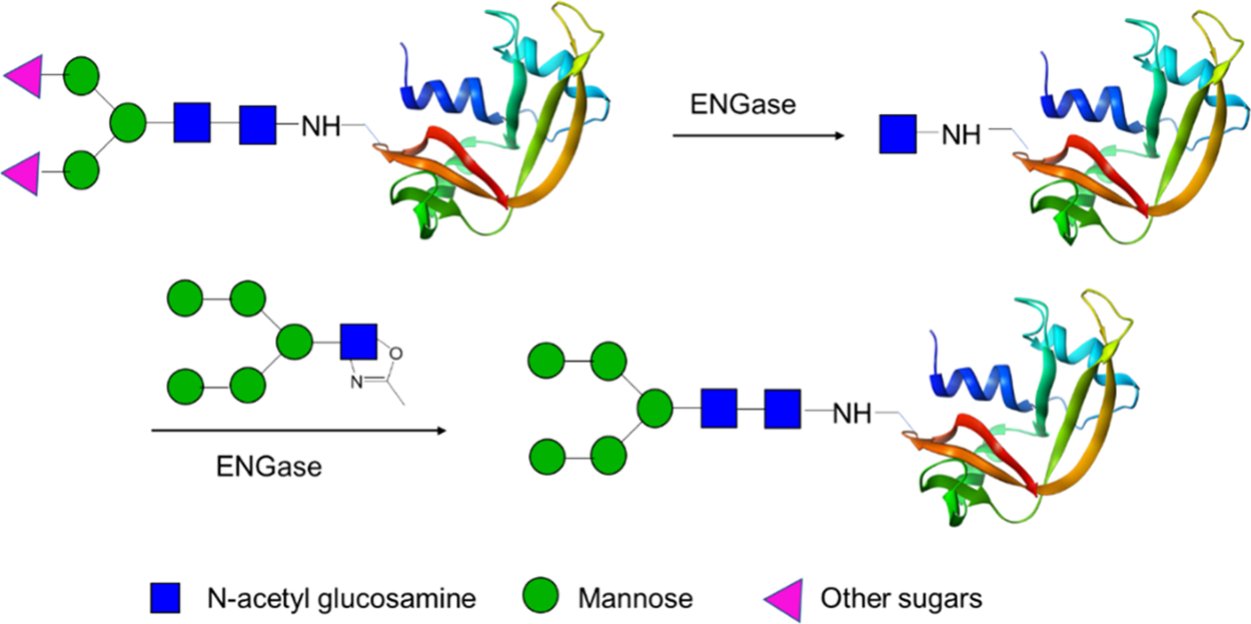
General Scheme for the Reaction Catalyzed by ENGases for the Remodeling of Sugar Moieties in Glycoproteins

Following this approach, an intriguing strategy in the development of GCVs could be the use of endoglycosidases to perform enzymatic glycosylation of antigenic proteins. *endo*-β-*N*-Acetylglucosaminidases (ENGases) naturally catalyze the cleavage of the chitobiose core of *N*-glycans between two GluNAcs but are also able to catalyze a glycosylation reaction to link a mannose glycan bearing an anomeric GluNAc to a peptide or protein glycated with N-linked GluNAc residues; sugars activated as oxazolines are the best donor substrates (Scheme 14).^67^

When glycosylation with a high-mannose oligosaccharide was attempted, ENGases showed high hydrolytic activity. Thus, variants were designed to obtain biocatalysts with reduced hydrolytic activity that could be suitable for the synthesis of products conjugated with complex oligosaccharides. The obtained mutant ENGases were confirmed to be effective in the glycosylation of different peptides derived from glycoprotein HIV-1 gp120^68^ and in the mannosylation of a 19 amino acid peptide derived from a human cytomegalovirus antigen.^69^

The introduction of α(1→3) galactose units at the end of oligosaccharide chains of inactivated viruses or subunit vaccines can also be a way to improve the antigen uptake, which in this case is mediated by α-Gal-immunoglobulins.^70^ Thus, with a recombinant α-1,3-GalT, the Galα(1→3)Galβ(1→4)GluNAc epitope on the hemagglutinin of the influenza virus was obtained by enzymatic glycosylation of *N*-linked lactosamine residues.

Epitope-specific αGal-immunoglobulins may be a target for oligosaccharide manipulation aimed at improving the activity of subunit vaccines. Indeed, the presence of sialic acid at the end of glycoproteins hampers their interaction with the antigen-presenting cell receptors that mediate the antigen uptake. Thus, remodeling of the carbohydrate chains of gp120 was performed with a one-pot enzymatic process involving a neuraminidase that removed sialic acid from the SAα(2→6)Gal(1→4)GluNAc-R chains to obtain *N*-acetyllactosamine residues. These were then the substrates for a recombinant α-1,3-GalT to form the α-Gal epitopes.^71^

This approach can be useful for enhancing the immunogenic activity of other glycoproteins in which the natural glycosylation “protects” the epitopes from the recognition of antigen-presenting cells.^72^ This is the case of the spyke protein of the coronavirus responsible for the SARS-CoV-2 pandemic.^73^

## Glycosyltransferases for the Chemoenzymatic Modification of Natural Antibiotics

The increasing diffusion of multidrug-resistant or extensively drug-resistant bacterial strains has driven our research toward the development of new antibiotics.^74^ However, the complexity of discovering,^4^ producing,^75,76^ and characterizing^53,77,78^ novel antibacterials limits the number of antibiotics that currently undergo full clinical development.

In this frame, the use of bioprocesses for the synthesis of novel sugar-based antibiotics may play a crucial role in extending the range of available therapeutic options. Indeed, microbial-derived antibacterials are frequently enzymatically regio- and stereospecifically glycosylated during their biosynthesis by the addition of unusual sugar moieties to polyketide or peptide chains, as in the case of macrolides and glycopeptides, respectively.^79^ In contrast, in aminoglycosides, polymerization of deoxyhexose and aminodeoxyhexose units originating from extensive enzyme-catalyzed modifications of common sugars directly generates the antibiotic scaffolds.^79^ Modifying the carbohydrate structures of existing antibiotics by a chemoenzymatic approach might enhance their antimicrobial potency, reduce side effects, and extend their activity towards resistant strains.

**Figure 2:**
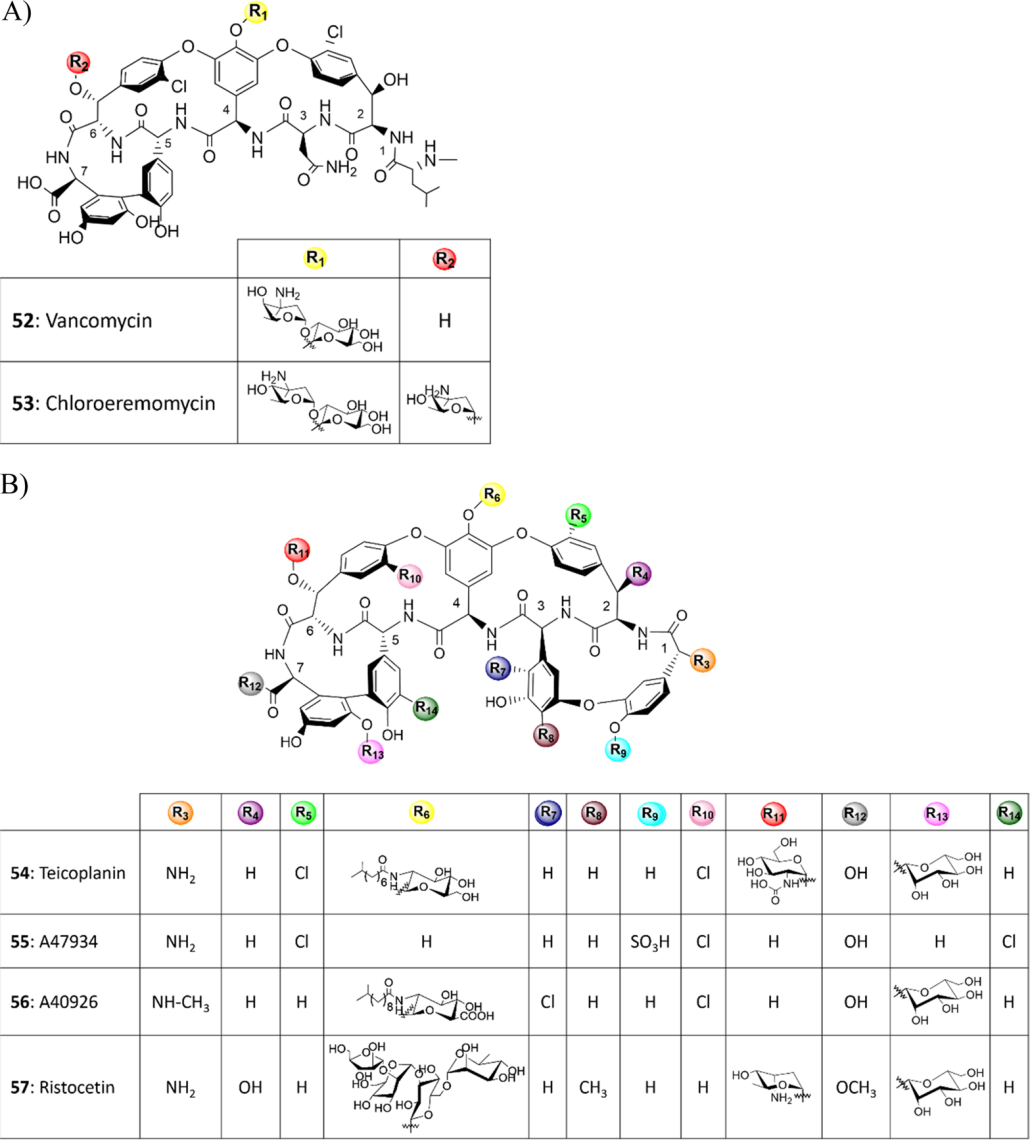
Structures of representative glycopeptide antibiotics.

In glycopeptide antibiotics (GPAs), the structure of heptapeptide aglycones is relatively conserved (**52**–**57**; Figure 2): their greatest diversity lies in the decoration pattern, which is defined by multiple enzymes encoded within the corresponding biosynthetic gene clusters (BGCs), including a variety of Gtfs.^4,80,81^ These have been used for the biocatalyzed modification of GPAs, as we recently reviewed.^8^

Other authors^82^ based their glycorandomization approach on chemical diversification of the sugars supplied to different Gtfs, generating several GPA variants, including some with enhanced biological activity against resistant strains. Gtfs may be promiscuous also in the nature of the heptapeptide aglycones used as substrates, although to different extents.^83^ Some examples are GtfE and GtfB that glycosylated amino acid 4 in vancomycin (**52**) and chloroeremomycin (**53**), respectively. In addition, GtfE could transfer a range of unnatural deoxy and amino sugars also to teicoplanin (**54**) and related aglycones, but GtfB poorly glycosylated these unnatural scaffolds.^83^

**Figure 3:**
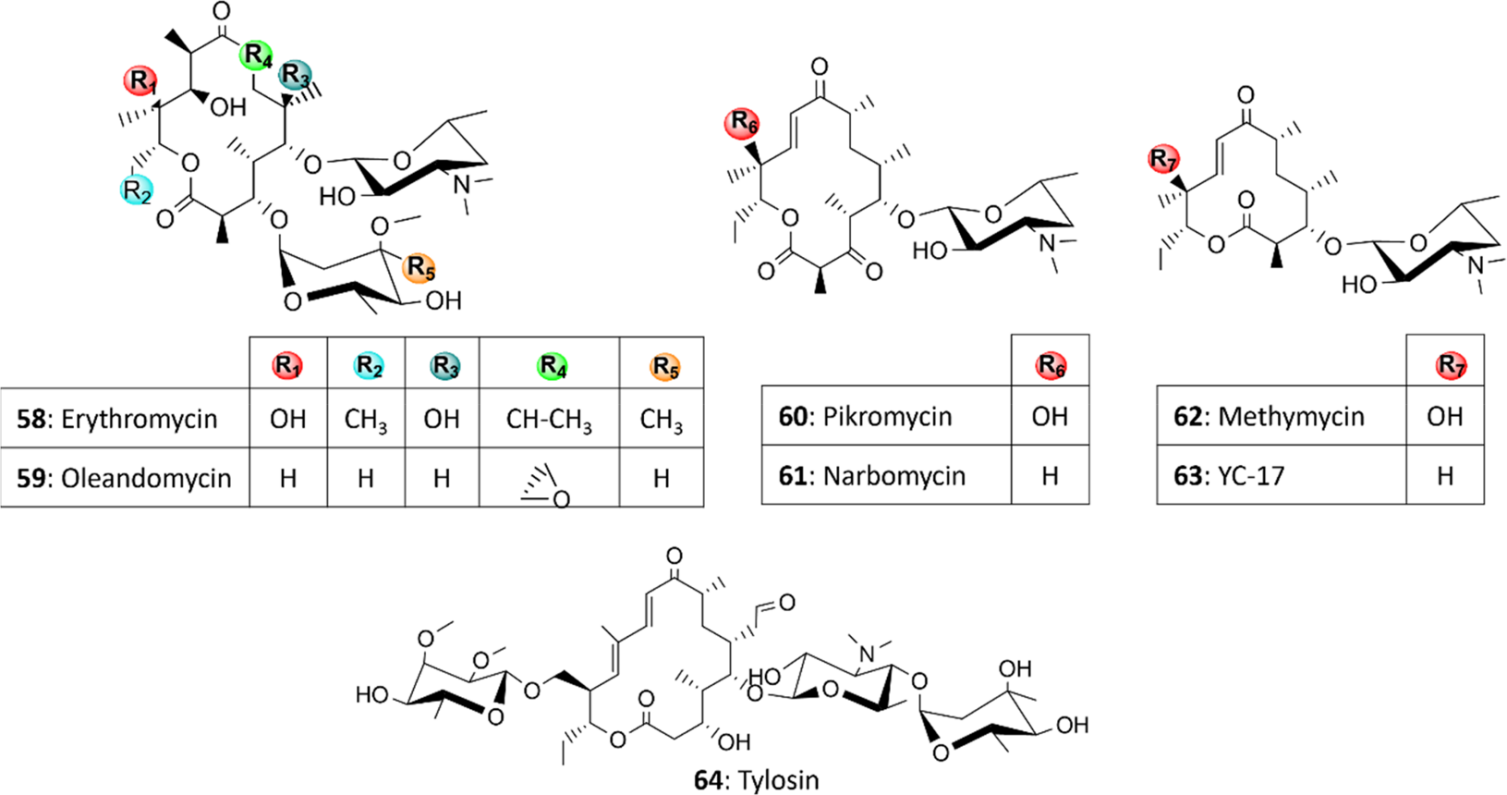
Structures of representative macrolide antibiotics.

Macrolide antibiotics are composed of a 14- or 16-atom macrolactone, produced through repeated condensation of acyl thioesters,^9^ which undergoes several tailoring reactions. Multiple deoxy sugars and amino sugars are linked to macrolactones by specific Gtfs, preferentially at C3 and C5, contributing to their biological activity (**58**–**64**; Figure 3).

The plasticity of macrolide Gtfs was demonstrated in several studies. For instance, the purified Gtf DesVII from *Streptomyces venezuelae*, the producer of methymycin (**62**), pikromycin (**60**), and its auxiliary protein DesVIII, were successfully used to transfer various 6-deoxy sugars, amino sugars, and *N*-alkylamino sugars to different macrolide aglycones (*e.g.*, deoxymethynolide and narbonolide) and their hydroxylated derivatives.^84^ Interestingly, the Gtfs OleD and OleI from oleandomycin (**59**)-producing *Streptomyces antibioticus* were used to glycosylate not only macrolides **59**, erythromycin (**58**), and tylosin (**64**) but also non-macrolide substrates such as flavanols, coumarins, and other aromatics, generating novel polyketide and coumarin antibiotics.^85^

**Figure 4:**
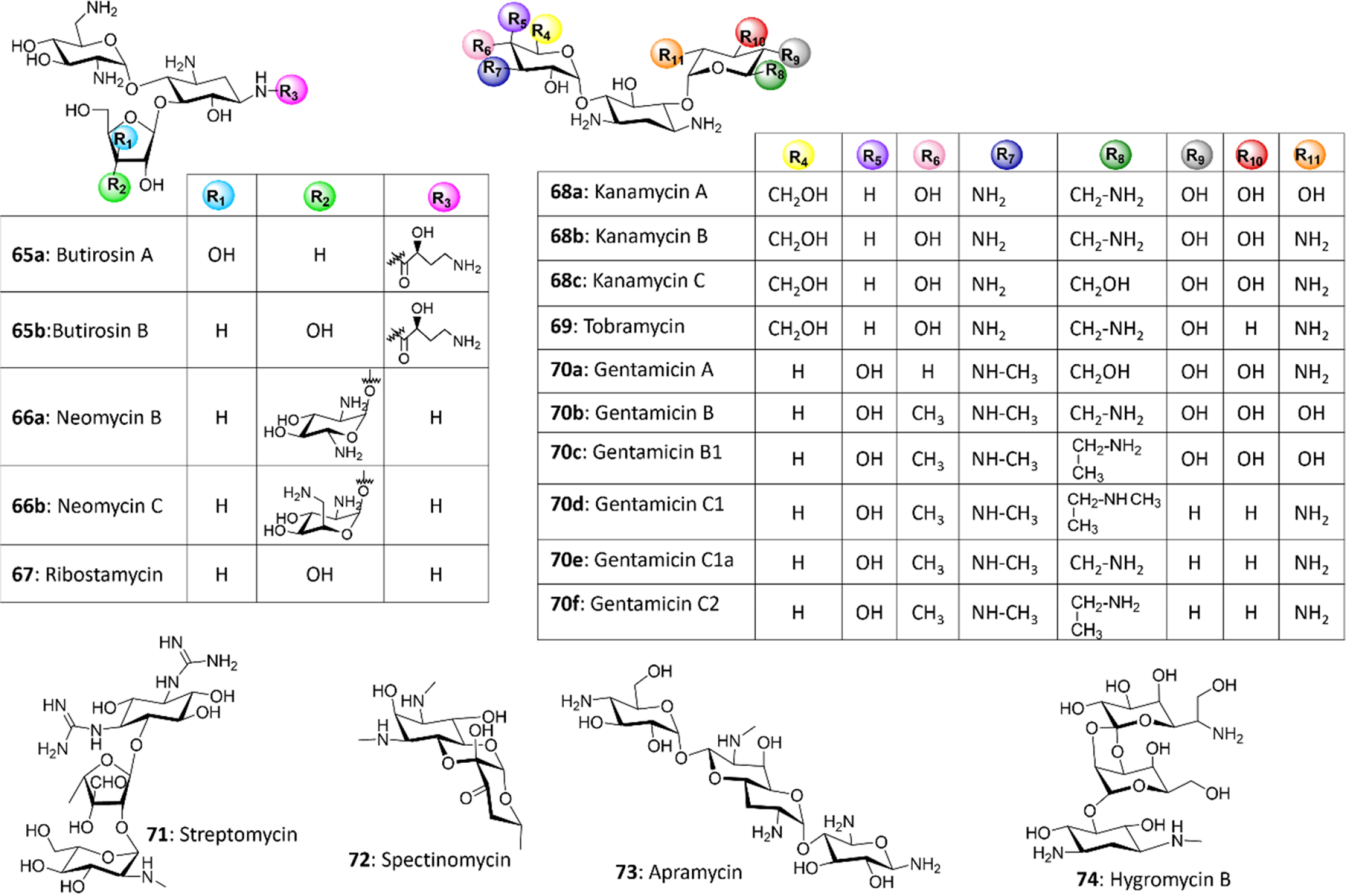
Structures of representative aminoglycoside antibiotics.

Aminoglycoside antibiotics are formed by a sugar-derived aminocyclitol decorated at C4, C5, and C6 with at least one amino and/or neutral sugar *viaO*-glycosidic linkages, with 2-deoxystreptamine (2-DOS) being the most common aminocyclitol.^10^ Aminoglycosides composed of 2-DOS are further divided into subgroups depending on the position of the sugar substituent: 4,5-disubstituted (**65**–**67**), 4,6-disubstituted (**68**–**70**), or monosubstituted at C4 or C5 (**71**–**74**) (Figure 4).

Multiple chemoenzymatic derivatization studies were conducted to modify existing aminoglycosides. For instance, 4,5-disubstituted 2-DOS derivatives were accepted as substrates by purified BrtH and BrtG, an acyltransferase and a γ-l-glutamyl cyclotransferase that are responsible for the attachment of the (*S*)-4-amino-2-hydrobutyrate (AHBA) side chain of butirosin (**65**) and for the removal of the protective γ-glutamyl group in the producer *B. circulans*, respectively. In this way, a series of AHBA-decorated aminoglycoside derivatives were produced.^86^

Constructing chimeric Gtfs by mix-and-match reprogramming represents an alternative and valuable solution for glycodiversification. Aminoglycoside derivatives were generated by the use of chimeric Gtfs produced by combining the N-terminal domain of kanamycin (**68**) Gtf KanF and the C-terminal module of **52** GtfE. Compared with the wild-type **68** Gtf, the most effective chimera catalyzed the glycosylation of 2-DOS with thiamine diphosphate (TDP)-d-glucose, guanidine diphosphate-d-mannose, and UPD-d-galactose with a significantly improved efficiency.^87^

It should be noted that besides the chemoenzymatic approaches described above, strategies of combinatorial biosynthesis are being more extensively used by many authors for the *in vivo* generation of novel antibiotics. For example, 15 diverse derivatives of the GPA A47934 (**55**) were generated by cloning the **55** BGC in the heterologous host *Streptomyces coelicolor* M1146 together with different combinations of 13 genes coding for tailoring enzymes from different GPA BGCs, leading to the production of glucosyl-, glucosaminyl-, and mannosyl derivatives of **55**.^88^ Hybrid macrolide antibiotics were instead generated *via* coexpression in a host organism of a deoxy sugar biosynthetic gene cassette together with genes encoding for Gtfs. As an example, when genes for TDP-d-desosamine synthesis were replaced in a mutant strain of the **62**/**60**-producer *S. venezuelae* YJ003 with gene cassettes for the sugars TDP-4-keto-6-deoxy-d-glucose, TDP-d-olivose, and TDP-d-quinovose, the above-cited DesVII/DesVIII were able to attach these non-native sugars both to endogenous macrolactones and to exogenously fed 12-, 14-, and 16-membered aglycones.^89,90^

## Conclusions and Perspectives

In this Account, we have reported the use of biocatalysis aimed at the sustainable synthesis of variegated carbohydrate-based drugs as anti-infective agents based on the current research in our laboratories. In view of the importance of glycosylated and glycoconjugated drugs and vaccines nowadays, further advances in this field are expected. In particular, we have described the use of hydrolases (*e.g.*, lipases, esterases, proteases, and glycosidases), glycosyltransferases, and enzymes involved in the synthesis of nucleoside derivatives (*e.g.*, nucleoside phosphorylases, *N*-deoxyribosyltransferases, and deoxynucleoside kinases). Different research groups have recently proposed the use of these enzymes for the synthesis of complex structures such as nucleoside derivatives, sugar polymer antigens, and glycoconjugate products such as glycoprotein vaccines, glycodendrimers, and glycocosylated antibiotics.

In addition, recent evolutions in *in vivo* combinatorial biosynthesis using microbial cells as recombinant biocatalysts are complementing the more classical chemoenzymatic approach for redesigning the structure of carbohydrate-containing antibiotics. The combination of advanced biocatalytic techniques (immobilization of enzymes, protein engineering, use of green solvents, and flow biocatalysis) with state-of-the-art green and synthetic chemistry methods are expanding our tools to produce a vast variety of glycosylated and glycoconjugated molecules ranging from small antibacterial and antiviral molecules to macromolecular glycodendrimers and GCVs. The interdisciplinary knowledge of these approaches will allow us in the near future to generate novel pathways for the biocatalyzed synthesis of carbohydrate-based drugs, with a positive impact on circular bioeconomy and public health.

## Abbreviations

AHBA: 4-amino-2-hydrobutyrate
AhPNP: PNP from *Aeromonas hydrophila*
AMM: arabinomannane mimetic
AXE: acetyl xylan esterase
BGC: biosynthetic gene cluster
CALB: lipase B from *Candida antarctica*
CpUP: UP from *Clostridium perfringens*
DNK: deoxynucleoside kinase
DdDAK: DNK from *Dyctiostelium discoideum*
DmDNK: DNK from the fruit fly *Drosophila melanogaster*
2-DOS: 2-deoxystreptamine
ENGase: *endo*-β-*N*-acetylglucosaminidase
GCV: glycovaccine
GluNAc: *N*-acetylglucosamine
GPA: glycopeptide antibiotic
Gtf: glycosyltransferase
HBV: hepatitis B virus
IME: iminomethoxy-ethyl
HIV: human immunodeficiency virus
IMER: immobilized enzyme reactor
MR: mannose receptor
NA: nucleoside analogue
NDP: nucleoside diphosphate
NDT: *N*-deoxyribosyltransferase
NP: nucleoside phosphorylase
PEI: poly(ethylenimine)
PNP: purine nucleoside phosphorylase
PSL-C: *Pseudomonas cepacia* lipase
ST: sialyl transferase
TDP: thiamine diphosphate
TP: thymidine phosphorylase
UDP: uridine diphosphate
UP: uridine phosphorylase
